# Outcome of Patients Treated Within and Outside a Randomized Clinical Trial on Neoadjuvant Chemoradiotherapy Plus Surgery for Esophageal Cancer: Extrapolation of a Randomized Clinical Trial (CROSS)

**DOI:** 10.1245/s10434-018-6554-y

**Published:** 2018-06-12

**Authors:** Eelke Toxopeus, Maartje van der Schaaf, Jan van Lanschot, Jesper Lagergren, Pernilla Lagergren, Ate van der Gaast, Bas Wijnhoven

**Affiliations:** 1 Department of Surgery, Erasmus MC – University Medical Center, Rotterdam, Rotterdam, The Netherlands; 2 Department of Molecular Medicine and Surgery, Karolinska University Hospital, 171 76 Stockholm, Sweden; 3 Division of Cancer Studies, King’s College London, London, UK; 4 Department of Medical Oncology, Erasmus MC – University Medical Center, Rotterdam, Rotterdam, The Netherlands

## Abstract

**Background:**

Randomized clinical trials (RCTs) can provide a high level of evidence for medical decision making, but it is unclear if the results apply to patients treated outside such trials.

**Objective:**

The aim of this study was to retrospectively compare outcomes of patients with esophageal cancer treated within and outside an RCT.

**Methods:**

All patients receiving neoadjuvant chemoradiotherapy (nCRT) plus surgery for esophageal cancer between 2002 and 2008 (ChemoRadiotherapy for Esophageal cancer followed by Surgery Study [CROSS] cohort) who participated in multicenter, phase II–III trials were compared with patients who underwent the same treatment outside the trial between 2008 and 2013 (post-CROSS cohort). The differences between these cohorts were analyzed using *t* tests, while logistic regression models were used to evaluate adverse events. Overall and disease-free survival were calculated using the Kaplan–Meier method and Cox regression analyses.

**Results:**

A total of 208 CROSS patients and 173 post-CROSS patients were included in this study. Patients from the post-CROSS cohort were older, had more co morbidities, and had poorer performance status. Clinical N stage, but not cT stage, was worse in the post-CROSS cohort. There were no statistically significant differences in adverse events (pulmonary, cardiac, or anastomotic complications) or survival between the comparison cohorts.

**Conclusion:**

The outcomes of patients treated with nCRT plus esophagectomy for cancer have a high external consistency and can be extrapolated to the daily practice of physicians involved in the treatment and care of esophageal cancer patients.

**Electronic supplementary material:**

The online version of this article (10.1245/s10434-018-6554-y) contains supplementary material, which is available to authorized users.

Randomized clinical trials (RCTs) can provide high levels of evidence for treatment efficiency in medicine;^1^, ^2^ however, RCTs often have strict inclusion and exclusion criteria, which might limit the generalizability of an RCT to a target population. The effectiveness and safety of treatment for a patient who does not match the eligibility criteria of trial participants is unclear.

Participation in an RCT, especially in the treatment arm, can be beneficial to patients.^3^ It is suggested that better care and closer and more frequent follow-up of trial participants might lead to better outcomes than in non-participants. Studies that have evaluated this question report mixed results.^4^–^7^

The ChemoRadiotherapy for Esophageal cancer followed by Surgery Study (CROSS) is an RCT that compared outcomes after neoadjuvant chemoradiotherapy (nCRT) plus surgery with surgery alone in patients with esophageal cancer.^8^–^10^ This study, as well as a meta-analysis, showed an improved survival of patients treated with nCRT.^11^ Hence, multimodality treatment is now considered standard of care for patients with resectable esophageal cancer. However, little is known about the selection and outcomes of patients receiving nCRT plus surgery in the setting of standard of care compared with patients who participated in the CROSS trial.

The aim of this study was to compare the patient characteristics and outcomes of CROSS study participants with patients who underwent nCRT plus surgery outside the study to evaluate whether outcomes remain similar.

## Methods

### Patients

Patients with histologically proven esophageal cancer who participated in the CROSS I and II studies that ran between February 2001 and January 2004 (CROSS I) and March 2004 through December 2008 (CROSS II) were defined as the CROSS cohort.^8^^,^^9^ Eight centers in The Netherlands participated in the CROSS trial. Inclusion and exclusion criteria have been reported previously.^9^^,^^10^ All patients with a resectable esophageal cancer (cT1N1 or T2–T4a, N0–N3, M0 tumor) and who were fit for nCRT plus surgery, as judged by the surgeon responsible, medical oncologist, and radiation oncologist between July 2008 and December 2013, were eligible for the post-CROSS cohort. These patients were all treated within the Erasmus MC. After the closure of patient recruitment for the CROSS trial, and before final publication of the full paper, the multidisciplinary team at the Erasmus MC had already considered nCRT as standard treatment based on systematic reviews. Hence, patients were treated from 2008 onwards outside the study protocol. These patients were identified from an institutional database (Erasmus MC – University Medical Centre Rotterdam). Ethical approval was not applicable because of the retrospective design of the study, as judged by the Ethical Committee of the Erasmus MC.

### Staging

All participating patients underwent history taking, physical examination, and routine hematological and biochemical tests. The standard tumor staging procedures included an upper gastrointestinal endoscopy with biopsies, endoscopic ultrasonography (EUS), and computerized tomography (CT) of the neck, chest and abdomen. EUS-guided fine-needle aspiration (FNA) was performed only when indicated; external ultrasonography of the neck, with FNA in case of suspected metastatic lymph nodes, and bronchoscopy and positron emission tomography (PET), were used in selected patients only. Only in T3 tumors was PET of any additional value at that time, and, in addition, was not yet standardized. Tumors were staged according to the tumor node metastasis (TNM) classification of the International Union Against Cancer 7th UICC-AJCC TNM staging manual.^12^

### Neoadjuvant Chemoradiotherapy

nCRT was administered within 5 weeks after completion of tumor staging and after discussion at the multidisciplinary team meeting. On days 1, 8, 15, 22, and 29, carboplatin and paclitaxel (targeted at an area under the curve of 2 mg/mL/min and at a dose of 50 mg/m^2^ of body surface area, respectively) were administered intravenously. Concurrently, external radiation was administered at a dose of up to 41.4 Gy in 23 fractions of 1.8 Gy each, with five fractions administered per week, starting on the first day of the first chemotherapy cycle.

### Surgery

For tumors involving the gastroesophageal junction, or in patients with a poor performance status (WHO performance score of 2 or higher), a transhiatal resection was preferred.^13^^,^^14^ A transthoracic approach with two-field lymph node dissection was mostly performed for tumors of the intrathoracic esophagus and for junctional tumors with positive lymph nodes at or above the carina. Dissection of the nodes along the celiac axis and its branches was performed in both approaches. A gastric tube reconstruction with cervical anastomosis was the preferred technique for restoring the continuity of the digestive tract. A minimally invasive approach was introduced in 2010, i.e. a thoracolaparoscopic esophagectomy (McKeown procedure) performed by a single surgeon. Lymph node dissection was similar to the open technique. An open left thoracoabdominal approach was used in some patients as part of a training program under the guidance of a teaching surgeon, and the resection specimen was evaluated for residual disease. Irradicality of the tumor resection margins (R1) was defined as vital tumor cells within 1 mm of the resection margins (proximal, distal and/or circumferential), and a pathologically complete response was defined as no vital tumor cells left in the resection specimen (ypT0N0M0), according to a modified Mandard score system.^15^^,^^16^

Complications were carefully registered for both cohorts; it is common sense to provide these data to the Dutch Upper GI Cancer Audit (DUCA).

### Follow-Up

All patients were seen in the outpatient clinic at least every 3 months during the first year after surgery and every 6 months during the second year, and were followed at least once a year in years 3, 4, and 5 after surgery. CT of the neck, chest and abdomen was only performed when a recurrence was clinically suspected.

### Statistical Analysis

Differences in patient characteristics between the comparison cohorts were assessed using the Student *t* test or Chi square test. When there were more than two categories within a parameter, a Chi square test for trend was used. The occurrence of adverse events was presented as frequencies, and differences in frequencies between the cohorts were calculated using the *t* test and presented as *p* values, with 5% as the level of statistical significance. Additionally, the odds of an occurrence of an adverse event in the two cohorts were calculated using univariable and multivariable logistic regression and presented as odds ratios (ORs) with 95% confidence intervals (CIs), and *p* values. The multivariable regression model included adjustment for potential confounding by age (continuous variable), sex, surgical approach (transhiatal or transthoracic), and tumor stage (categorized according to the 7th TNM classification Ia, Ib, IIa, IIb, IIIa–IIIb, IIIc and IV). The probability of survival over time was estimated using the Kaplan–Meier method, and the log-rank test was used to assess differences between the cohorts. All patients were updated in July 2016 with regard to date of recurrence, survival, and last day of follow-up. To determine variables that affected survival, a Cox regression model was used to calculate hazard ratios (HRs) with 95% CIs, with adjustment for age, sex, surgical approach, complications (categorized as any or none), and tumor stage.

## Results

### Patients

A total of 208 patients (51 from CROSS I and 157 from CROSS II) were included in the CROSS cohort, while the post-CROSS cohort consisted of 173 patients. Patients in the post-CROSS cohort were older and had more comorbidities and poorer performance status. In addition, clinical N stage, but not cT stage, was worse in the post-CROSS cohort (Table 1).

**Table 1:** Patient and tumor characteristics of 381 patients, divided into the CROSS (*n* = 208) and post-CROSS (*n* = 173) cohorts for patients with oesophageal or junctional cancer who underwent neoadjuvant chemoradiotherapy according to CROSS followed by surgery

		CROSS	Post-CROSS	
		*N* (%)	*N* (%)	*p* value
Total		208	173	
Age, years	Mean [SD]	60 [0.8]	62 [0.7]	0.004
	<60	107 (51)	62 (36)	0.001
	60–65	37(18)	36 (21)	
	66–69	35 (17)	33 (19)	
	70–75	27 (13)	28 (16)	
	> 75	2 (1)	14 (8)	
Sex	Male	163 (78)	137 (79)	0.8
	Female	45 (22)	36 (21)	
Comorbidity	No	162 (78)	113 (65)	0.002
	One or more	46 (22)	60 (35)	
Charlson index	0	162 (78)	110 (64)	0.007
	1	40 (19)	48 (28)	
	2	6 (3)	14 (8)	
	3			
Karnofsky performance status	60	1 (0)	0 (0)	0.000
	70	2 (1)	0 (0)	
	80	9 (4)	16 (9)	
	90	90 (44)	126 (73)	
	100	73 (35)	8 (5)	
	Missing	33 (16)	23 (13)	
Tumor length, cm	Mean [SD]	4.5 [0.15]	5.1 [0.19]	0.008
	≤ 8	183 (86)	154 (91)	
	> 8	7 (4)	16 (9)	0.02
	Missing	18 (9)	3 (2)	
Clinical T stage	T1	1 (0)	9 (5)	0.04
	T2	30 (14)	37 (21)	
	T3	176 (85)	115 (66)	
	T4	0 (0)	8 (5)	
	Missing	1 (0)	4 (2)	
Clinical N stage	N0	78 (37)	53 (31)	0.000
	N1	114 (55)	54 (31)	
	N2	13 (6)	55 (31)	
	N3	2 (1)	8 (4)	
	Missing	1 (0)	3 (2)	
Histology	Adenocarcinoma	160 (77)	133 (76)	0.5
	Squamous cell carcinoma	48 (23)	40 (23)	

*CROSS* ChemoRadiotherapy for Oesophageal cancer followed by Surgery Study, *SD* standard deviation

Fourteen patients who underwent nCRT did not proceed to surgery because of poor general condition or as a result of the patient’s own decision. In another 19 patients, distant dissemination was present at surgical exploration or the primary tumor or lymph nodes found could not be resected. These patients were excluded from the final analyses.

### Treatment Characteristics and Pathology

More than 95% of patients in each cohort finished all five cycles of neoadjuvant chemotherapy and 23 fractions of radiotherapy, and there were no statistically significant differences in completion rate between the cohorts (*p* = 0.348 and *p* = 0.196, respectively). The mean (standard deviation) time between the end of nCRT and surgery was 6.6 weeks (0.1) for the CROSS cohort and 7.9 weeks (0.3) for the post-CROSS cohort (*p* < 0.001) (Table 2). Pathological tumor stage was not statistically significantly different between the cohorts (*p* = 0.76). The percentage of patients with complete pathological response (ypT0N0M0) was 27% (*n* = 56) in the CROSS cohort and 28% (*n* = 49) in the post-CROSS cohort (*p* = 0.76).

**Table 2:** Details on treatment regimen and pathological assessment of the resection specimen of 381 patients, divided into the CROSS (*n* = 208) and post-CROSS (*n* = 173) cohorts for patients with oesophageal or junctional cancer who underwent chemoradiotherapy according to CROSS followed by surgery

		CROSS	Post-CROSS	
		*N* (%)	*N* (%)	*p* value
Total		208	173	
Chemotherapy	< 5 cycles	4 (2)	6 (4)	0.348
	5 cycles	204 (98)	167 (96)	
Radiotherapy	< 23 cycles	2 (1)	0 (0)	0.196
	23 cycles	206 (99)	173 (100)	
Weeks between end of nCRT and surgery	Mean [SD]	6.6 [0.1]	7.9 [0.3]	< 0.001
	≤ 6	95 (46)	48 (28)	< 0.0001
	> 6	113 (54)	125 (72)	
Surgical approach	Transthoracic	92 (44)	89 (52)	0.734
	Transhiatal	116(56)	56 (33)	
	Other^a^	0 (0)	28 (16)	
Resection margins	R0	195 (94)	159 (92)	0.486
	R1	13 (6)	14 (8)	
ypT stage^b^	T0	71 (34)	57 (33)	0.65
	T1	29 (14)	25 (14)	
	T2	41 (20)	30 (17)	
	T3	64 (31)	60 (35)	
	T4	2 (1)	1 (1)	
	Missing	1 (0)	0 (0)	
ypN stage^b^	N0	144 (69)	108 (62)	0.22
	N1	45 (22)	51 (29)	
	N2	15 (7)	9 (5)	
	N3	4 (2)	5 (3)	
LN ratio	Mean [SD]	0.065 [0.142]	0.046 [0.092]	< 0.0001
Pathological complete response^c^	T0N0M0	56 (27)	49 (28)	0.76
Differentiation grade	Poor	26 (12)	53 (31)	< 0.0001
	Moderate	26 (12)	49 (28)	
	Good	1 (1)	3 (2)	
	Unknown, including pCR	155 (75)	68 (40)	

*CROSS* ChemoRadiotherapy for Oesophageal cancer followed by Surgery Study, *nCRT* neoadjuvant chemoradiotherapy, *SD* standard deviation, *R0* tumor-free resection margin, *R1* tumor cells within 1 mm or at the resection margin, *ypT stage* T stage after nCRT, *ypN stage N* stage after nCRT, *pCR* pathologically complete response, *LN ratio* ratio of positive/resected lymph nodes divided by the number of resected lymph nodes (between 0 and 1)

^a^Other approaches, including minimally invasive esophagectomy and left thoracoabdominal approach

^b^Pathological T and N stage after neoadjuvant chemoradiotherapy

^c^Pathologically complete response (ypT0N0M0)

### Adverse Events

There were no statistically significant differences in adverse events (pulmonary, cardiac, or anastomotic complications) between the cohorts, except for chylothorax (see electronic supplementary material). The OR of infectious complications was increased in the post-CROSS cohort compared with the CROSS cohort (OR 1.88, 95% CI 0.99–3.58, *p* = 0.054), but the difference was not statistically significant (electronic supplementary material).

### Survival

Median overall survival was 44.2 months (interquartile range [IQR] 15.2–64.9); median overall survival in the CROSS cohort was 58.5 months (IQR 19.0–86.8) versus 35.0 months (IQR 12.9–51.4) in the post-CROSS cohort (95% CI 16.1–29.4).

**Fig. 1:**
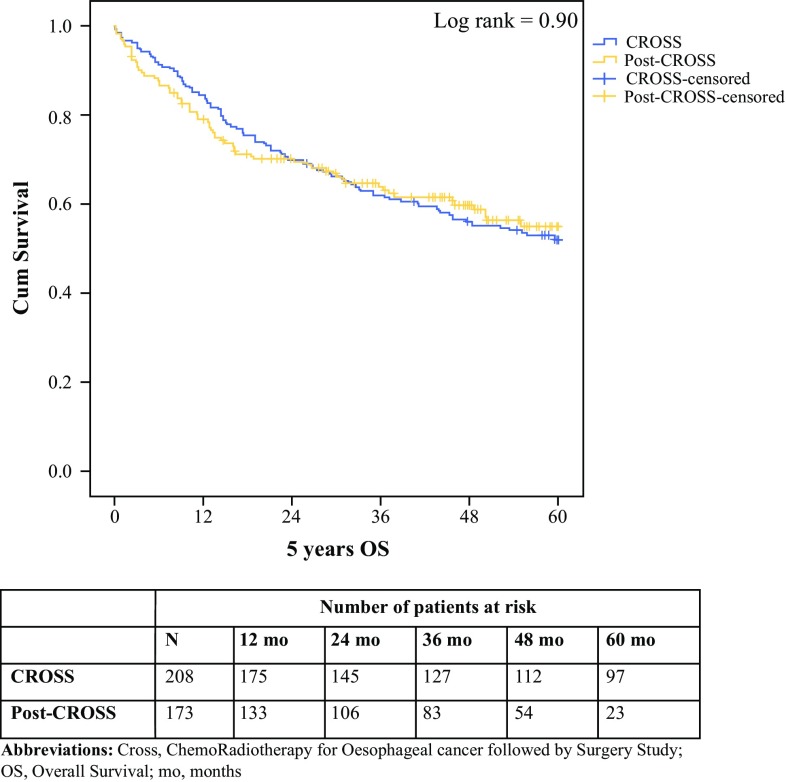
Overall survival of 381 patients, divided into the CROSS (*n* = 208) and post-CROSS (*n* = 173) cohorts for patients with oesophageal or junctional cancer who underwent chemoradiotherapy according to CROSS followed by surgery (*p* = 0.90). *cum survival* percentages of cumulative overall survival, where 1.0 means 100% of the cohort, decreasing over time, *5-year OS* 5-year overall survival, expressed in months, *CROSS* ChemoRadiotherapy for Esophageal cancer followed by Surgery Study, *mo* months

**Fig. 2:**
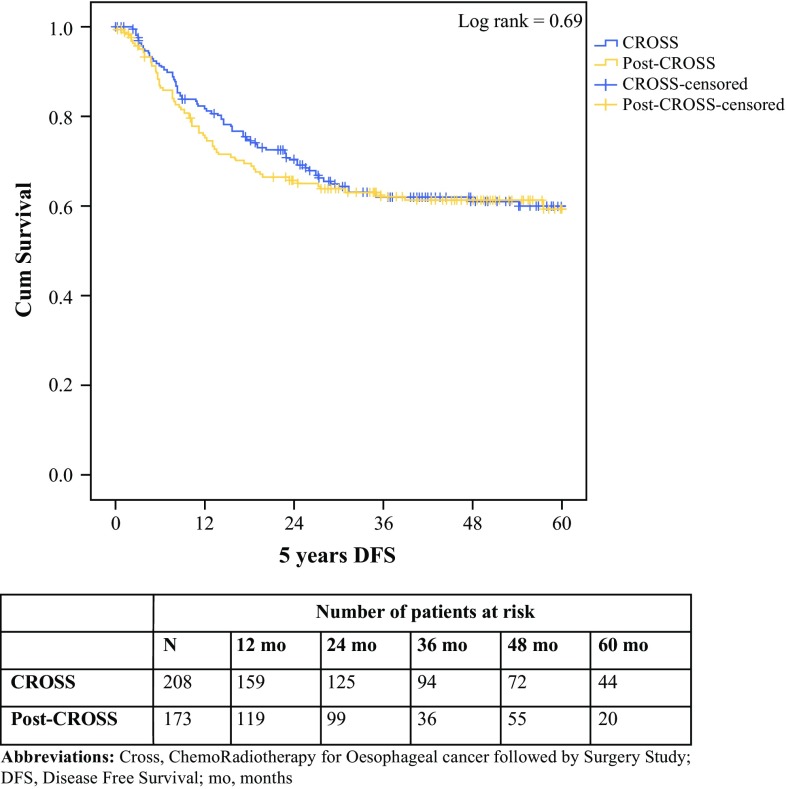
Disease-free survival of 381 patients, divided into the CROSS (*n* = 208) and post-CROSS (*n* = 173) cohorts for patients with oesophageal or junctional cancer who underwent chemoradiotherapy according to CROSS followed by surgery (*p* = 0.69). *cum survival* percentages of cumulative disease-free survival, where 1.0 means 100% of the cohort, decreasing over time, *5*-*year DFS* 5-year disease-free survival, expressed in months, *CROSS* ChemoRadiotherapy for Esophageal cancer followed by Surgery Study, *mo* months

The HRs of mortality were similar when comparing cohorts for overall survival, 30- and 90-day mortality, and disease-free survival (Table 3). Overall 5-year survival and 5-year disease-free survival were not statistically significantly different between the CROSS and post-CROSS cohorts (log-rank 0.90, overall 95% CI 39.2–43.8; and log-rank 0.69, overall 95% CI 39.6–44.5, respectively) (Figs. 1, 2).

**Table 3:** Hazard ratios for mortality comparing patients who underwent CROSS inside a trial (reference) with patients treated in the post-CROSS era

	HR	95% CI	*p* value
30-day mortality	1.37	0.40–4.68	0.62
90-day mortality	0.53	0.23–1.25	0.15
Overall survival	1.02	0.75–1.39	0.90
Disease-free survival	0.93	0.67–1.31	0.69

*CROSS* ChemoRadiotherapy for Oesophageal cancer followed by Surgery Study, *HR* hazard ratio, *CI* confidence interval

## Discussion

This study shows similar survival rates in patients included in the CROSS trial and those treated after the RCT, after adjustment for confounders. Those who underwent nCRT plus surgery outside the CROSS trial were older and had more comorbidities and a poorer performance status. In addition, more patients with T1 tumors, as well as patients with extensive nodal disease (cN3 stage), underwent multimodality treatment as the inclusion criteria of the CROSS trial excluded these patients. This may indicate that the multidisciplinary team has become more liberal in selecting patients for nCRT, given the confirmed effectiveness of this treatment.^9^^,^^10^

The poorer performance status of patients in the post-CROSS cohort did not translate into a decreased tolerance to nCRT. In both cohorts, more than 95% of patients completed the treatment and went on to have surgery. The toxicity profile of the CROSS regimen is favorable compared with other neoadjuvant regimens, including the MAGIC regimen and 5-fluorouracil/cisplatin combination.^8^^,^^17^ This could have also played a role in the decision of the multidisciplinary team to also recommend nCRT for older and frail patients.^18^ Age alone is not considered an absolute contraindication for surgery with or without neoadjuvant treatment.

Overall survival and disease-free survival were not statistically significantly different between the cohorts. In addition, postoperative complications did not differ, despite the small difference in comorbidity and performance status of the cohorts. A non-significantly higher percentage of patients in the post-CROSS cohort underwent a transthoracic resection, which did not seem to translate into more pulmonary or cardiac complications, as has been previously reported.^13^^,^^18^ Pathological tumor stage was also not significantly different between the cohorts, which supports a high efficacy of the multimodal treatment that persisted in the years after the trial finished. Since the publication of the CROSS study, nCRT is used by more institutes in the Southwest of the Netherlands, including centers that refer patients to the Erasmus MC for surgery. The fact that a pathologically complete response is obtained in a large percentage of patients in the post-CROSS cohort is indicative of the sustained efficacy of the chemoradiotherapy treatment.

The time to surgery after finishing nCRT was somewhat longer for the post-CROSS cohort, which reflects logistic problems in the center and a less-stringent planning of the operation, as is usually dictated by a study protocol. A longer time to surgery may affect pathological staging but might not impact on survival, as shown in the present study.^19^

Enrolment in RCTs may lead to better outcomes in patients with cancer. In chronic myelocytic leukemia, the survival rate of patients within clinical trials was higher than patients outside trials.^20^ An explanation for this could be the access to better medications and, in particular, the selection of healthier patients for trials. In a recent paper on surgery for a benign upper gastrointestinal disease, trial participation did not affect clinical outcome.^4^

One of the limitations of this present study is that the post-CROSS cohort was retrospectively evaluated, which may have introduced bias and incomplete reporting of outcome parameters, including complications such as toxicity of the nCRT; increased toxicity in elderly patients with poorer performance status and more comorbidity may have been missed. However, overall survival and mortality are unambiguous endpoints. It should be noted that follow-up of the post-CROSS cohort was not as long as the CROSS cohort. Nevertheless, the recurrence of esophageal cancer typically occurs within 2 years of surgery and most patients were followed up for more than 24 months. Changes in surgical techniques (minimally invasive techniques) and perioperative patient management could, to some extent, have influenced outcomes in favor of the post-CROSS cohort. Moreover, selection bias could have occurred since patients who did not receive nCRT in the years after publication of the CROSS study could not be identified.

Another weakness of this study is that both cohorts may not be completely similar due to the fact that the CROSS cohort, acting as the control, was derived from a randomized controlled trial, wherein a variety of hospitals participated. The post-CROSS cohort was identified, in retrospect, from a single tertiary referral center (also participating in the original CROSS study). Furthermore, the inclusion criteria for patients receiving nCRT were expanded on. Although no formal changes in the perioperative care protocol (e.g. enhanced recovery program) took place at the Erasmus MC during the study period, minor changes in perioperative care, field planning for radiotherapy, and time between the end of nCRT and surgery may have occurred, with a (small) impact on the (short-term) outcomes reported in this study,^18^^,^^21^ It is unlikely that overall survival, the main outcome measure of this study, is affected by these factors. Finally, some tumors could not be restaged retrospectively from the TNM 6th edition (CROSS I and II) to the TNM 7th edition,^12^ which may have had a small impact on the CROSS cohort in relation to N stage.

When the inclusion criteria of the CROSS trial were projected onto the patients of the post-CROSS cohort, 14 patients did not qualify for nCRT due to older age, and 19 patients had a tumor length of > 8 cm. Despite this finding, it is felt that while there are differences in patient and tumor characteristics between the two cohorts, it is safe to apply nCRT to most patients with a resectable esophageal cancer who have been evaluated and discussed in a multidisciplinary team. In these patients, the benefit in survival and harm of the multimodal treatment is likely within the same range, as reported in patients participating in the CROSS trial.

## Conclusion

The outcomes of patients treated with nCRT plus esophagectomy for esophageal cancer have a high external consistency and can possibly be extrapolated to the daily practice of physicians involved in the treatment and care of esophageal cancer patients.

## Electronic Supplementary Material

Below is the link to the electronic supplementary material.

Supplementary material 1 (DOCX 12 kb)
