# Supplementary material for: Outcome of Patients Treated Within and Outside a Randomized Clinical Trial on Neoadjuvant Chemoradiotherapy Plus Surgery for Esophageal Cancer: Extrapolation of a Randomized Clinical Trial (CROSS)
Source: Ann Surg Oncol. 2018 Jun 12;25(8):2441–8. doi: 10.1245/s10434-018-6554-y (PMC6029046; doi:10.1245/s10434-018-6554-y)
Supplement: Supplementary file 1 — Supplementary material 1 (DOCX 12 kb) [file 10434_2018_6554_MOESM1_ESM.docx]

**Adverse events after surgery (supplementary data).**Adverse events after surgery of 381 patients, divided in a CROSS (N=208) and Post CROSS (N=173) cohort with patients with oesophageal or junctional cancer who underwent chemoradiotherapy according to CROSS followed by surgery.

|  | CROSS  (n=208) | Post CROSS  (n=173) |  |
| --- | --- | --- | --- |
|  | **Number (%)** | **Number (%)** | **P-value** |
| Any adverse event | 132 (64) | 109 (63) | 0.83 |
| Pulmonary complication | 93 (45) | 66 (38) | 0.17 |
| Cardiac complication | 39 (19) | 27 (16) | 0.40 |
| Anastomotic complication | 46 (22) | 49 (28) | 0.18 |
| Infectious complication | 18 (9) | 20 (12) | 0.36 |
| Chylothorax | 4 (2) | 10 (6) | 0.05 |
| 30-day mortality | 6 (3) | 5 (3) | 1.00 |

**Abbreviations:** CROSS, CROSS, ChemoRadiotherapy for Oesophageal cancer followed by Surgery Study.

**Odds for adverse events after surgery for patients in the CROSS and the post-CROSS cohort (supplementary data).**

|  | OR* | 95% CI | P-value |
| --- | --- | --- | --- |
| Any complication | 0.87 | 0.56-1.36 | 0.55 |
| Pulmonary complications | 0.73 | 0.47-1.12 | 0.15 |
| Cardiac complications | 0.60 | 0.33-1.07 | 0.08 |
| Anastomotic complications | 1.30 | 0.80-2.10 | 0.28 |
| Infection | 1.88 | 0.99-3.58 | 0.05 |
| Chylothorax | 3.24 | 0.97-10.82 | 0.06 |

**Abbreviations:** CROSS, ChemoRadiotherapy for Oesophageal cancer followed by Surgery Study; OR, Odds ration; CI, Confidence Interval.

*Adjusted for age, sex, tumour stage, surgical approach and comorbidity
